# Exploring behavioral intention to use telemedicine services post COVID-19: a cross sectional study in Saudi Arabia

**DOI:** 10.3389/fpubh.2024.1385713

**Published:** 2024-04-16

**Authors:** Raniah N. Aldekhyyel, Faisal Alshuaibi, Osama Alsaaid, Faisal Bin Moammar, Talal Alanazy, Abdulmajeed Namshah, Kholood Altassan, Reem Aldekhyyel, Amr Jamal

**Affiliations:** ^1^Medical Informatics and E-learning Unit, Medical Education Department, College of Medicine, King Saud University, Evidence-Based Health Care & Knowledge Translation Research Chair, Riyadh, Saudi Arabia; ^2^College of Medicine, King Saud University, Riyadh, Saudi Arabia; ^3^Department of Family & Community Medicine, College of Medicine, King Saud University, Riyadh, Saudi Arabia; ^4^Department of English Literature, College of Languages, Princess Nourah Bint Abdulrahman University, Riyadh, Saudi Arabia; ^5^Evidence-Based Health Care & Knowledge Translation Research Chair, Family & Community Medicine Department, King Saud University, Riyadh, Saudi Arabia

**Keywords:** telemedicine, attitudes, behavioral intention, eHealth literacy, post COVID-19, Saudi Arabia

## Abstract

**Introduction:**

While telemedicine offers significant benefits, there remain substantial knowledge gaps in the literature, particularly regarding its use in Saudi Arabia. This study aims to explore health consumers’ behavioral intention to use telemedicine examining the associated factors such as eHealth literacy and attitudes toward telemedicine services.

**Methods:**

A cross-sectional observational study was conducted to collect data on demographics, health status, internet skills, attitudes toward telemedicine, and eHealth literacy. An online survey was administered at two large public gatherings in Riyadh. The eHEALS-Pl scale was used to measure perceived eHealth literacy levels, and data analysis was performed using SPSS (IBM Corp. United States).

**Results:**

There were 385 participants, with an equal distribution of genders. The largest age group was 18–20 years old (57%). Nearly half of the participants were neither employed nor students, while 43% had access to governmental hospitals through employment. 71% reported proficiency in using the internet. Health-wise, 47% rated their health as excellent, and 56% did not have medical insurance. 87% expressed a high likelihood of using telemedicine if offered by a provider. Participants were categorized based on their eHealth Literacy scores, with 54% scoring low and 46% scoring high. Overall, participants showed positive attitudes toward telemedicine, with 82% agreeing that it saves time, money, and provides access to specialized care. About half of the participants perceived the process of seeing a doctor through telemedicine video as complex. Both eHealth Literacy and attitudes toward telemedicine showed a statistically significant association with the intention to use telemedicine (*p* < 0.001). There was a positive and significant correlation between eHealth Literacy and attitudes (*ρ* =0.460; *p* < 0.001). Multivariate ordinal regression analysis revealed that the odds for a high likelihood of intention to use telemedicine significantly increased with positive attitudes (*p* < 0.001). Mediation analysis confirmed the significant mediating role of attitudes toward telemedicine in the relationship between eHealth Literacy and the intention to use telemedicine.

**Conclusion:**

The findings underline the importance of enhancing health literacy and consumer attitudes toward telemedicine, particularly during the healthcare digital transformation we are experiencing globally. This is crucial for promoting increased acceptance and utilization of telemedicine services beyond the COVID-19 pandemic.

## 1 Introduction

In recent years, significant advancements have been made in the field of healthcare, closely paralleling the rapid progress in technology. One notable breakthrough is the emergence of telemedicine services. Telemedicine encompasses the utilization of technology, such as video conferencing, to facilitate remote medical consultations, diagnosis, and treatment (1). Recognized by the American Telemedicine Association for its potential to enhance healthcare accessibility, reduce costs, and improve patient satisfaction (2), telemedicine has been viewed as a promising solution to address healthcare delivery challenges, particularly for individuals residing in remote areas or with limited access to medical services (3–6). However, despite its notable benefits, the adoption of telemedicine services has been hindered by low utilization (7–9).

Numerous studies have investigated the factors influencing the adoption of telemedicine services in both developed and developing countries (7–12). These determinants encompassed patients’ acceptance and trust, healthcare providers’ attitudes, regulatory frameworks, and technological infrastructure. It is imperative to address these factors to ensure successful implementation of telemedicine services.

Saudi Arabia, a country experiencing rapid population growth expected to reach 39 million by 2050 (13), could greatly benefit from using telemedicine services. Although the country has a well-developed healthcare system that includes both public and private facilities, access to medical services is still limited, especially in rural or remote regions (14). The Ministry of Health is the primary government provider and funder of healthcare in Saudi Arabia, operating hospitals and primary health care (PHC) centers. These services constitute 60% of the country’s total health services. Other government entities, including referral hospitals, security forces medical services, army forces medical services, National Guard health affairs, Ministry of Higher Education hospitals, ARAMCO hospitals, Royal Commission for Jubail and Yanbu health services, school health units of the Ministry of Education, and the Red Crescent Society, provide services to specific populations, such as employees and their dependents, with some offering services during crises and emergencies. The private sector also plays a role in healthcare delivery, especially in urban areas, with several hospitals and clinics (15, 16). Studies investigating access to PHC centers in the country have highlighted various constraints, such as inequitable access to health services, distance to clinics, the growing burden of chronic diseases, and infrastructure limitations (17, 18). A potential solution to bridge this gap is the utilization of telemedicine services (19). Recent developments in Saudi Arabia have shown promising advancements in telemedicine, including the establishment of the Saudi Seha-virtual hospital, a state-of-the-art platform that provides remote healthcare services to patients across the country (20). The Saudi Seha-virtual hospital has the potential to overcome geographical barriers and enhance healthcare accessibility by offering teleconsultations, remote diagnosis, and treatment options. These developments indicate a growing recognition of the importance of telemedicine in the Saudi healthcare landscape and the commitment to overcome existing barriers.

Recent studies have investigated the potential use of telemedicine services in Saudi Arabia. Alghamdi et al. assessed healthcare providers’ perspectives on telemedicine during the COVID-19 pandemic, finding moderate to high levels of readiness (21). AlBar et al. studied patients’ acceptance of eHealth services, noting the significant influence of perceived usefulness and ease of use on attitudes. However, they highlighted the need for increased patient awareness and education regarding eHealth services, which can affect behavioral intention (22). Al-Samarraie et al. conducted a systematic review exploring barriers and facilitators to telemedicine implementation in Middle Eastern countries, including Saudi Arabia. They identified barriers such as religious and social restrictions, resistance to change, and literacy levels, which influence individuals’ perceptions and attitudes toward accepting telemedicine (23). Overcoming these barriers is crucial for the successful implementation of telemedicine services in the country.

Despite the potential advantages of telemedicine, there are notable knowledge gaps in the literature, particularly concerning Saudi Arabia. Existing research on telemedicine in the country has primarily centered on user perception, acceptance, or satisfaction. However, it is crucial to comprehend health consumer attitudes and behavioral intentions, defined by the Theory of Reasoned Action/Theory of Planned Behavior as “the amount of effort one is willing to exert to attain a goal” (24), regarding the use of telemedicine services. This understanding is essential for ensuring the effective utilization of telemedicine services and promoting healthcare accessibility. Further investigation is necessary to understand public perceptions, attitudes, and intentions to use telemedicine as a mode of healthcare delivery, especially in a post-pandemic era. This can be achieved by adopting behavioral intention models, such as the Health Information Technology Behavioral Intention Model (25). This model provides a framework for understanding how individuals’ attitudes, perceived usefulness, and perceived ease of use influence their behavioral intentions to use health information technology, including telemedicine services. Guided by this model, we can obtain valuable insights into the utilization of telemedicine in Saudi Arabia, including public attitudes and intentions to adopt telemedicine and the factors influencing acceptance, is crucial for healthcare providers and policy makers.

To address these gaps, our goal was to investigate the behavioral intention of health consumers in Saudi Arabia toward using telemedicine services and explore associated factors such as eHealth literacy and attitudes toward telemedicine services, drawing on similar factors examined by Ghaddar et al. (26). Our study aimed to achieve the following objectives: (1) assess the attitudes of Saudi citizens toward telemedicine services and their level of eHealth literacy, (2) examine factors influencing behavioral intentions to utilize telemedicine services, and (3) investigate the impact of attitudes and eHealth literacy on behavioral intentions.

## 2 Materials and methods

### 2.1 Study design, population and sampling technique

This research employed a cross-sectional observational design and enrolled participants through convenience sampling. The inclusion criteria consisted of Saudi citizens aged 18 years and older, irrespective of their demographic backgrounds, genders, educational levels, occupations, and geographic locations. The minimum age requirement of 18 years was established to ensure that participants had the legal capacity to provide informed consent and actively engage in the study. Sample size estimation was conducted using the Raosoft sample size calculator (27), considering a 5% margin of error and a 95% confidence level. The calculation determined that a minimum of 377 survey responses was necessary to obtain reliable results.

### 2.2 Study instrument development and validation

The research survey’s items were adopted from a validated survey by Ghaddar et al. (26) and modified to align with the research setting and objectives of the study. After the research team conducted modifications, the survey underwent a rigorous review process involving domain experts. The survey was then translated into Arabic by a proficient bilingual translation expert (R.A) and reviewed by the research team, applying a team-based collaborative and iterative translation approach (28). To test the validity and reliability of the survey, a pilot study was conducted with a sample of 25 participants. Feedback obtained during this stage was instrumental in finalizing the research survey.

The research survey included five sections: (1) demographic data and background; (2) health status; (3) internet skills; (4) attitudes toward telemedicine; and (5) eHealth literacy. The survey was developed using Google Forms (29), was in Arabic only, and took approximately 7–10 min to be completed.

### 2.3 Outcome measure

In this study, we evaluated the outcome measure of “behavioral intention to use telemedicine services” (Intention) using a specific question: “How likely are you to use telemedicine services if your provider offered them?” Participants selected their responses from a range of options; “very likely,” “somewhat likely,” and “not very likely.”

### 2.4 Predictor measures

There were five predictor measures in the study: sociodemographic variables, health status, internet skills, attitudes toward telemedicine (Attitudes), eHealth literacy scale (eHEALS).

#### 2.4.1 Demographic characteristics and background

We collected sociodemographic variables, which included age, gender, region, employment status, education level, yearly household income, and insurance coverage. To measure health status, participants were asked to evaluate their health condition using a 3-point Likert scale (excellent, good, bad) and to answer a yes/no question about whether they suffer from a chronic disease. To measure participants internet skills, participants were asked a yes/no question about their own perception regarding their skill level “Do you think you are skilled in using the Internet (skills to search and extract relevant content from the Internet and evaluate the information extracted)?”

#### 2.4.2 Attitudes toward telemedicine

To measure attitudes toward telemedicine, participants rated their level of agreement on a 5-point Likert scale (1 = strongly disagree to 5 = strongly agree) with nine statements reflecting different aspects of telemedicine use, such as perceived ease of use, perceived usefulness, and perceived cost-effectiveness, originally developed by Guruper et al. (30) and modified by Ghaddar et al. (26). The Arabic version of the attitudes toward telemedicine scale, which ranged from 9 to 45 with higher scores indicating more positive attitudes, showed good internal consistency (Cronbach’s alpha = 0.788), above the acceptable threshold value of 0.70 (31). In comparison to Ghaddar et al.’s study (26), Cronbach alpha, assessing the internal consistency of the attitudes scale for the Spanish surveys was 0.757, while the original English surveys was 0.839.

#### 2.4.3 eHealth literacy score (eHEALS)

To measure eHealth literacy, the eHealth Literacy Scale (eHEALS) (32) was used, where respondents indicated their level of agreement on a 5-point Likert scale (1 = strongly disagree to 5 = strongly agree) for each item. The total score, ranging from 8 (lowest possible eHealth literacy) to 40 (highest possible eHealth literacy) was calculated by summing the responses. In line with previous studies utilizing the eHEALS scale (33, 34), we employed the median value as a cut-off to classify participants into high and low eHealth Literacy groups. This approach has also been consistently discussed by methodologists over time (35). Cronbach alpha, assessing the internal consistency of eHEALS for our sample, was 0.926 which was substantial.

### 2.5 Data collection

Data collection took place in Riyadh, Saudi Arabia, at two prominent public venues, the Riyadh International Book Fair and Riyadh Front, over a three-week period in June 2022. These venues were strategically selected as central hubs that attract individuals from diverse socioeconomic backgrounds across the Kingdom. To ensure a more representative sample of the general population, data were collected at different times and on different days. Similar settings, such as U.S state fairs, have been utilized (36). Individuals present at the venues during various times of the day were approached by the data collection research team. They were briefed about the study’s objectives and invited to participate. Those meeting the inclusion criteria and expressing interest received a QR code, guiding them to the web-based survey. Throughout the survey completion, the research team remained available to address any questions participants may have had.

### 2.6 Ethical consideration

This study received ethical approval from the Institutional Review Board (IRB) of King Saud University (E-22-7046). Informed consent was obtained from all participants prior to their participation. The collected data were anonymized, and confidentiality was maintained by securely storing all research data in a password-protected file accessible only to the research team.

### 2.7 Data analysis

Data analysis was performed using SPSS version 29 (Armonk, NY: IBM Corp. USA). Continuous variables were summarized as means ± standard deviation (SD) or median and interquartile range (IQR); while categorical variables were summarized using frequency and percentages. The normality of the variables was assessed using the Kolmogorov–Smirnov test. A Chi-square test or a Fisher’s exact test was used to test the association between intention to use telemedicine, with other categorical variables. Kruskal Wallis test, which is a non-parametric alternative to one way ANOVA, was employed to compare differences between groups of continuous variables. An ordinal regression analysis was conducted. Variables achieving statistical significance in the univariate analysis were included in the multivariate regression analysis. A mediation analysis, using the macro called PROCESS v4.2 by Andrew F. Hayes., was employed to test the mediation effect of Attitudes in assessing the relationship between eHEALS and the Intention. The Intention was classified as low and high likelihood while performing the mediation analysis. Significance was set at *p* < 0.05.

## 3 Results

### 3.1 Demographic characteristics and background

A total of 385 participants took part in the study, comprising approximately 51% females and 49% males, with the majority falling in the 18–20 age group (57%). Most participants reported a yearly household income of less than 50,000SR (43%). The majority reported coming from the middle region (80%), with nearly half not employed or students, and about 43% having access to governmental hospitals through employment. Health-wise, 85% reported no chronic illnesses, 47% rated their health as excellent, and 56% lacked medical insurance. Approximately 71% indicated proficiency in internet usage. Most participants (87%) expressed a high likelihood of using telemedicine services if offered by a healthcare provider (Table 1).

**Table 1:** Participant demographics.

Characteristic	*N*	%
Age in years (median (Inter Quartile Range))	27.00 (23.0, 35.0)	
18–28	219	56.9
29–39	113	29.4
40–50	41	10.6
>51	12	3.1
Gender		
Male	187	48.6
Female	198	51.4
Participant Region		
North	14	3.6
East	16	4.2
Middle	309	80.3
West	20	5.2
South	26	6.8
Employment status		
Employed	199	51.7
Non-employed	83	21.6
Self-employed	22	5.7
Student	81	21.0
Education		
High school	89	23.1
Diploma	38	9.9
College Degree	227	59.0
Post Graduate Degree	31	8.1
House-hold income per year		
<50,000 SR	164	42.6
50,000–100.000 SR	77	20.0
100,000–150,000 SR	65	16.9
>150,000 SR	79	20.5
Health insurance coverage		
No	217	56.4
Yes	168	43.6
Access to Hospitals through employment		
Yes, government hospital	164	42.6
Yes, private hospital	110	28.6
No access	111	28.8
Do you think you are skilled in using the internet?		
Yes	275	71.4
Some what	110	28.6
Assessment of health		
Excellent	319	82.9
Good	64	16.6
Bad	2	0.5
Chronic illness		
No	325	84.4
Yes	60	15.6
Intention of using telemedicine		
High likelihood (*very likely*)	333	86.5
Intermediate likelihood (*somewhat likely*)	42	10.9
Low likelihood (*not very likely*)	10	2.6

A Chi square or a Fisher’s exact test was used to examine the association between the demographic variables, insurance coverage, and access to hospitals with the intention to use telemedicine services. None of these variables showed a statistically significant association with Intention.

### 3.2 Attitudes toward telemedicine

**Figure 1:**
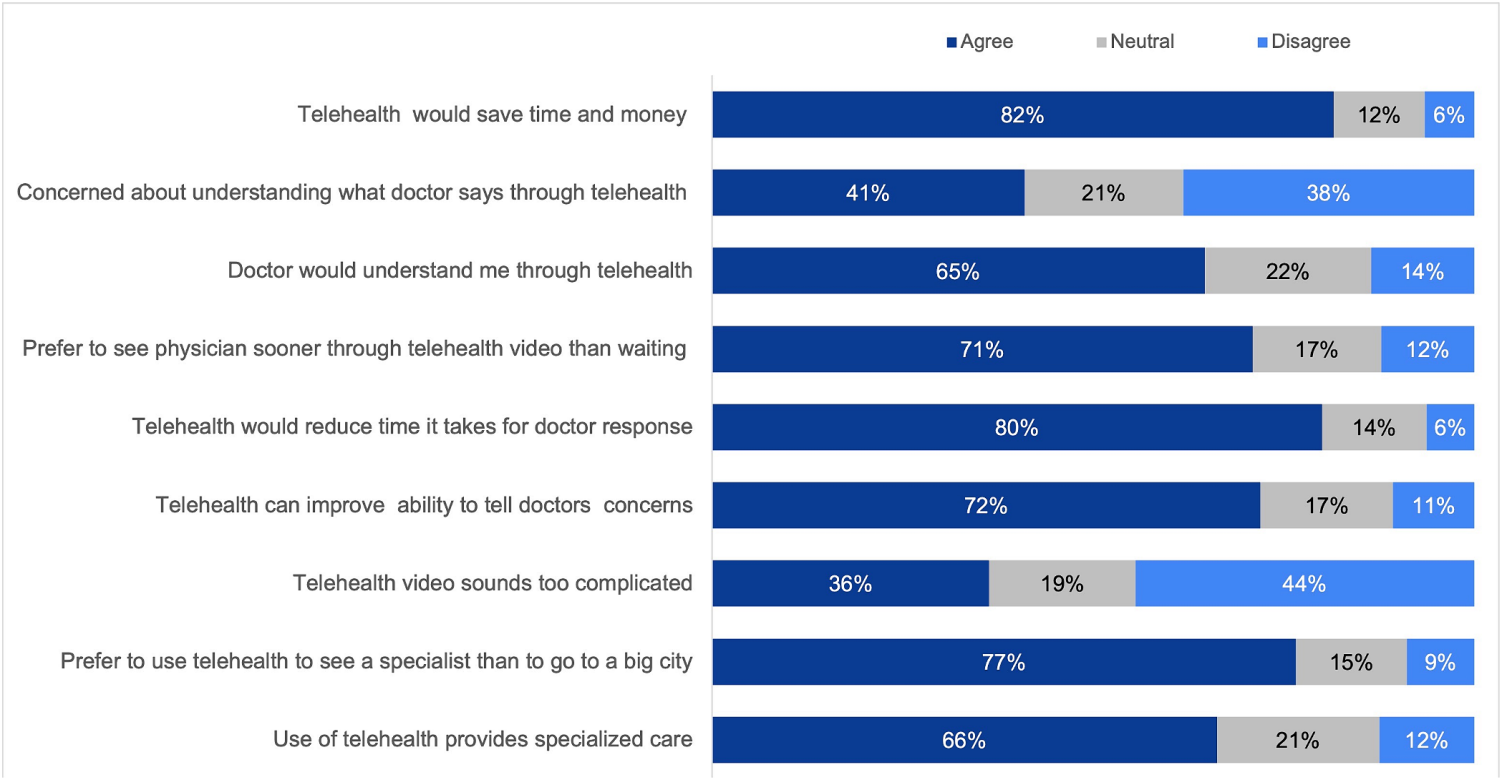
Attitudes toward telemedicine.

For analysis purposes, the response choices “strongly agree” and “agree” were combined into a single category labeled “agree.” Similarly, the response choices “strongly disagree” and “disagree” were combined into a single category labeled “disagree.” Overall, participants conveyed positive attitudes toward the adoption of telemedicine. A substantial majority, comprising 82%, agreed that telemedicine could save time and money while providing access to specialized care. However, a noteworthy area of concern was identified among half of the participants, particularly related to the perceived complexity of consulting with a doctor through telemedicine video (Figure 1).

### 3.3 eHealth literacy score (eHEALS)

To determine low and high eHealth literacy scores, the median value of 30 was employed. The sample was then divided into two groups: those with a low eHealth literacy score (median 30 and below) and those with a high score (median above 30). In this study, around 43% of respondents achieved a low eHEALS score, while 46% obtained a high eHEALS score. A Chi square or a Fisher’s exact test was used to examine the association between eHEALS, internet skills, and health status with the intention to use telemedicine services. None of these variables showed a statistically significant association with Intention, except for eHEALS, which showed a significant association with Intention (*p* < 0.001) (Table 2).

**Table 2:** The correlation between Intention to use telemedicine with eHEALS using the Spearmen’s rank correlation test.

Characteristic	Intention to use telemedicine			*p*-value
	Low likelihood *n* (%)	Intermediate likelihood *n* (%)	High likelihood *n* (%)	
eHEALS category				
Low eHEALS_P1 score	36 (17.4)	84 (40.6)	87 (42.0)	0.001
High eHEALS_P1 score	16 (9.0)	52 (29.2)	110 (61.8)	

### 3.4 Association between variables

A Kruskal-Wallis test provided very strong evidence of a difference (*p* < 0.001) between the mean ranks of at least one pair of groups of Intention with eHealth literacy. Dunn’s pairwise tests were carried out for the three pairs of groups. There was very strong evidence (*p* < 0.001, adjusted using the Bonferroni correction) of a difference between the group with low Intention and intermediate Intention with high Intention. The median score of eHealth literacy for the group with low Intention was 27.0 compared to 31.0 in the high Intention group. The score of eHealth literacy of the group intermediate Intention was 29.0. There was no evidence of a difference between the low Intention and intermediate Intention groups for eHealth literacy. High likelihood of Intention group was significantly different from the low and the intermediate likelihood groups in their median eHealth literacy score (Table 3).

**Table 3:** Results of Kruskal-Wallis test and the *post hoc* Dunn’s multiple comparisons test, showing significance of differences in intention with eHealth literacy and attitudes.

Characteristic	Kruskal Wallis Test		Dunn’s multiple comparison test		
	H	*p*	1–2	1–3	2–3
			*p*	*p*	*p*
eHealth literacy	22.727	0.001	ns	0.001	0.001
Attitudes toward telemedicine	69.425	0.001	0.001	0.001	0.001

ns, non-significant; 1–2 (Low likelihood vs Intermediate likelihood); 1–3 (Low likelihood vs High likelihood); 2–3 (Intermediate likelihood vs High likelihood).

The Kruskal Wallis test showed evidence of the difference in the ranks of Attitudes between the different groups of Intention. Dunn’s pairwise tests showed a statistically significant difference between all the three pairs of groups, with median score of Attitudes being 30.0, 36.0 and 38.0, respectively, for the low, intermediate, and high levels of Intention. All the three groups were significantly different in their median Attitudes scores with the high likelihood of Intention group scoring the highest median score.

**Figure 2:**
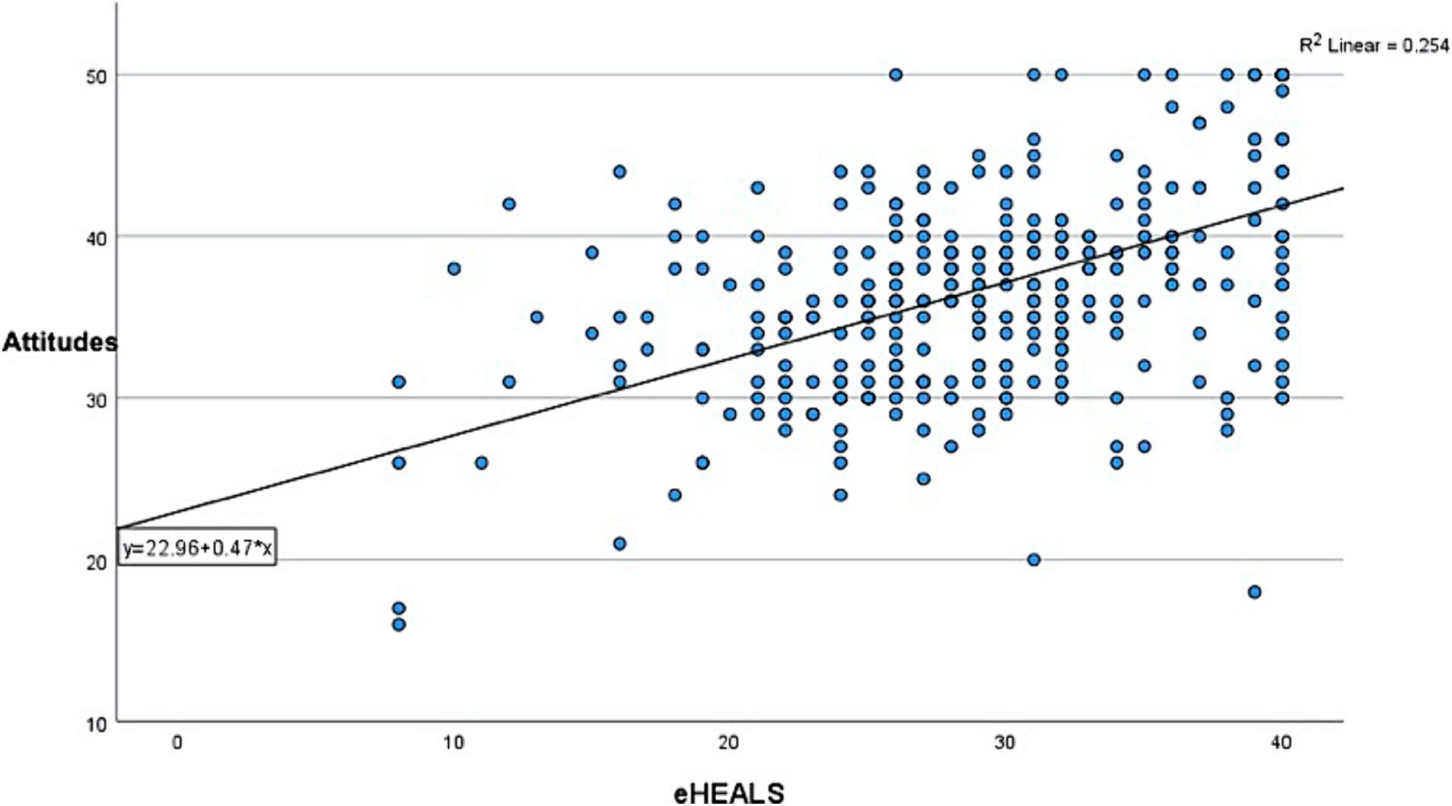
Correlation between eHealthy literacy scores and attitudes score.

The correlation between eHealth literacy scores and Attitudes scores was positive and statistically significant suggesting a positive, linear relationship between the two (*ρ* = 0.460; *p* < 0.001) (Figure 2). A multivariate ordinal regression analysis showed that the odds for higher likelihood of telemedicine use was 0.145 times more likely to increase with increasing Attitudes score, which was statistically significant (*p* < 0.001). But the relationship between eHealth literacy and Intention was not statistically significant, suggesting a probable mediation effect of Attitudes on the relationship between eHealth literacy and Intention (Table 4). Control variables were not included in the model since none of the variables were statistically significant, and their inclusion did not significantly improve or change the variability explained by the model.

**Table 4:** Multivariate ordinal regression model of factors associated with Intention to use telemedicine services.

Characteristics	Coefficient (SE)	95% CI	Odds Ratio (95% CI)	*p*
eHealth literacy	0.006 (0.017)	(−0.027–0.040)	1.00 (0.97–1.04)	0.721
Attitudes toward telemedicine	0.145 (0.021)	(0.105–0.186)	1.16 (1.11–1.20)	0.001

**Figure 3:**
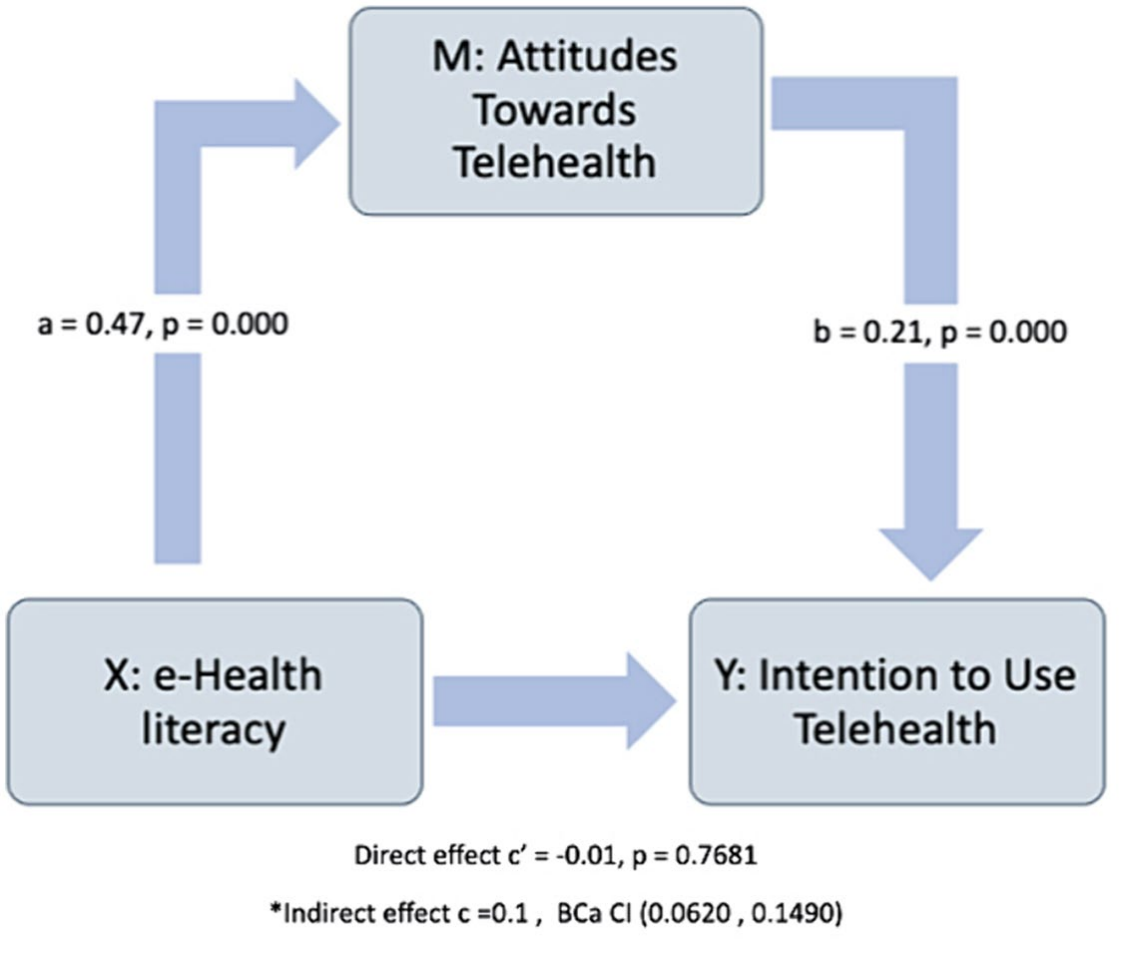
Representation of the mediation analysis performed. The relationship between the predictor (eHealth literacy) and the outcome (Intention) is denoted by path c. The eHealth literacy also predicts the mediator (Attitudes toward telemedicine). *The confidence interval for the indirect effect is a BCa bootstrapped CI based on 5,000 samples.

Figure 3 shows the diagrammatic representation of the mediation analysis performed. The relationship between the predictor (eHealth literacy) and the outcome (Intention) is denoted by path c. The eHealth literacy also predicts the mediator (Attitudes toward telemedicine) denoted by path a, Attitudes predicts Intention denoted by path b. The path c’ represents the relationship between eHealth literacy and the Intention in the presence of a mediator. The analysis shows a significant effect of the mediator on the relationship between the predictor and the outcome. The effect of eHealth literacy on Attitudes was significant firstly, denoted by path *a* = 0.47, *p* < 0.001. The relationship between Attitudes and Intention was also significant denoted by path *b* = 0.21, *p* < 0.001. The direct relationship between eHealth literacy and Intention was not significant *c*’ = −0.01, *p* = 0.7681, but there was a significant indirect effect of eHealth literacy on Intention through Attitudes, *c* = 0.1. The mediating effect of Attitudes likely reduced the strength of the relationship between eHealth literacy and intention to use telemedicine (Table 5).

**Table 5:** Mediation analysis of attitudes on the effect of eHealth literacy and intention.

Characteristics	Coefficient (SE)	*p*
eHealth literacy (a)	0.47 (0.04)	0.0001
Attitudes (b)	0.21 (0.04)	0.0001
eHealth literacy (direct effect c’)	−0.01 (0.03)	0.7681
eHealth literacy (indirect effect c)	0.1 (0.02)*	(0.06, 0.15)*

*estimates from 5,000 bootstrapped samples.

## 4 Discussion

Since the start of the COVID-19 pandemic in 2020, there has been a significant surge in the implementation of telemedicine services in healthcare systems globally. Originally employed to ensure the uninterrupted delivery of care during the pandemic, many healthcare organizations have sustained, enhanced, and established telemedicine as a method of care delivery. Recognizing the continuing use of telemedicine beyond the constraints of the pandemic, it becomes vital to understand the various factors shaping health consumers’ intentions to continue utilizing telemedicine services post-COVID-19. This understanding is crucial for healthcare providers and policymakers in advancing healthcare delivery.

This study examined the behavioral intentions of health consumers in Saudi Arabia regarding the utilization of telemedicine services. The research explored the association between Intentions, consumers’ Attitudes, and their eHealth literacy. Overall, participants in our study demonstrated positive attitudes toward telemedicine usage. A significant majority expressed agreement with the notion that telemedicine can save time and money while providing access to specialized care. However, a noteworthy concern for more than half of the participants was related to the perceived complexity of consulting a doctor through telemedicine video. A finding that was not shared by other studies conducted in the Middle East Region (12, 37, 38). Most of our research population were under the age of 28, without insurance, and rely on governmental health organizations for their healthcare needs. The observed challenges with the perceived complexity of telemedicine platforms might arise from the lack of user-friendly interfaces (39, 40) or a potential gap in consumer awareness on how to effectively use telemedicine services (41). Given the crucial role of governmental organizations in accommodating the needs of the population, it becomes essential to enhance the user experience of their telemedicine platforms, especially considering Saudi Arabia’s substantial investment in digital healthcare transformation as part of the 2030 Saudi Vision. To address the potential gap in consumer awareness, implementing approaches to ensure that every patient accessing telemedicine services for the first time receives comprehensive instructions on platform usage is needed. Designing tailored training programs, considering the technology and health literacy levels of health consumers, presents another effective approach to bridge these gaps (42).

Our study found a significant association between eHEALS and participants’ Intention to utilize telemedicine services. Specifically, health consumers with high eHEALS scores demonstrated a stronger intention toward utilizing telemedicine, given that the younger population was the majority represented in our study. This finding emphasizes the critical role of eHealth literacy in shaping consumer behaviors toward telemedicine, which is consistent with the findings in other studies conducted globally (42–46). A strong understanding of digital health concepts, combined with proficient skills in navigating online health resources, appears to have the potential to promote telemedicine adoption. As such, efforts aimed at enhancing eHealth literacy among the population may be beneficial in promoting the widespread acceptance and utilization of telemedicine services. Additionally, this association highlights the importance of incorporating eHealth literacy assessments into telemedicine implementation strategies, ensuring that health consumers are adequately equipped to adopt the full potential of digital health technologies, including telemedicine services (42).

The impact of eHealth literacy on attitudes toward telemedicine was found to be statistically significant in our analysis. Moreover, we observed a significant relationship between Attitudes and Intentions to use telemedicine services. Although the direct association between eHealth literacy and Intention was not statistically significant, our analysis revealed a significant indirect effect of eHealth literacy on Intention through Attitudes. This suggests that while eHealth literacy may not directly influence health consumers’ intentions to use telemedicine, it employs an indirect influence through shaping their Attitudes toward telemedicine. Health consumers’ attitudes toward telemedicine likely play a role in decreasing the strength of the relationship between their eHealth literacy levels and their Intentions to utilize telemedicine services. These findings were consistent with the finding published by Ghaddar et al. (26).

This study holds significant implications for advancing our understanding of the influence of health consumers’ attitudes toward telemedicine and eHealth literacy on consumers’ intention to use telemedicine services, especially in understudied populations like Saudi Arabia. By measuring eHealth literacy in this specific context, the research contributes valuable insights into the factors shaping consumers’ intention to adopt telemedicine services when offered. The findings underline the importance of enhancing health literacy, particularly during the healthcare digital transformation we are experiencing globally, to facilitate greater acceptance and utilization of telemedicine service.

When discussing the potential limitations of our study, it’s crucial to acknowledge the reliance on self-reported data, which may introduce response bias. Additionally, our study unintendedly focused on the perceptions of the younger population living in Riyadh, the capital city of Saudi Arabia. Future research should aim to include participants living in rural areas and from an older population to provide a more comprehensive understanding of telemedicine adoption. While we assessed the relationship between eHealth literacy, attitudes, and intentions toward telemedicine, other factors such as cultural beliefs, privacy concerns, and past experiences with telemedicine were not explored. These factors may have influenced participants’ perceptions and should be considered in future studies (47).

Future research could investigate the effectiveness of interventions aimed at enhancing eHealth literacy and shaping positive attitudes toward telemedicine. The development of tailored public health interventions and awareness programs structured as effective methods to increase health literacy, may have the potential to promote the use of telemedicine services. Longitudinal studies tracking individuals’ experiences with telemedicine over time could provide valuable insights into the factors that contribute to the attitudes and intentions toward telemedicine adoption. Comparative studies across different regions and cultural contexts would enrich our understanding of the factors influencing telemedicine acceptance and utilization beyond the COVID-19 pandemic.

## Data availability statement

The raw data supporting the conclusions of this article will be made available by the authors, without undue reservation.

## Ethics statement

The studies involving humans were approved by Institutional Review Board (IRB) of King Saud University (E-22-7046). The studies were conducted in accordance with the local legislation and institutional requirements. The participants provided their written informed consent to participate in this study.

## Author contributions

RNA: Conceptualization, Data curation, Formal analysis, Funding acquisition, Investigation, Methodology, Supervision, Validation, Visualization, Writing – original draft, Writing – review & editing. FA: Conceptualization, Data curation, Formal analysis, Investigation, Project administration, Writing – original draft, Writing – review & editing. OA: Conceptualization, Data curation, Formal analysis, Investigation, Project administration, Writing – original draft, Writing – review & editing. FB: Conceptualization, Data curation, Formal analysis, Investigation, Project administration, Writing – original draft, Writing – review & editing. TA: Conceptualization, Data curation, Formal analysis, Investigation, Project administration, Writing – original draft, Writing – review & editing. AN: Conceptualization, Data curation, Formal analysis, Investigation, Project administration, Writing – original draft, Writing – review & editing. KA: Methodology, Validation, Writing – original draft, Writing – review & editing. RA: Methodology, Validation, Writing – original draft, Writing – review & editing. AJ: Conceptualization, Methodology, Validation, Writing – original draft, Writing – review & editing.
